# *Enterococcus faecium* R0026 Combined with *Bacillus subtilis* R0179 Prevent Obesity-Associated Hyperlipidemia and Modulate Gut Microbiota in C57BL/6 Mice

**DOI:** 10.4014/jmb.2009.09005

**Published:** 2020-10-20

**Authors:** Jinli Huang, Juan Huang, Tianyi Yin, Huiyun Lv, Pengyu Zhang, Huajun Li

**Affiliations:** 1 Department of Microecology, College of Basic Medical Sciences, Dalian Medical University, Dalian 6044, P.R. China; 2 First Affiliated Hospital of Dalian Medical University, Dalian, P.R. China

**Keywords:** Adiposity, probiotics, microflora, inflammation, serum lipids

## Abstract

*Bacillus subtilis* and *Enterococcus faecium* are commonly used probiotics. This study aimed to identify the effect of live combined *Bacillus subtilis* R0179 and *Enterococcus faecium* R0026 (LCBE) on obesityassociated hyperlipidemia and gut microbiota in C57BL/6 mice. Forty male C57BL/6 mice were divided into four groups: normal group (N group), model group (M group), low-dose group (L group), and high-dose group (H group). Mice were gavaged with LCBE at 0.023 g/mice/day (L group) or 0.23 g/mice/day (H group) and fed with a high-fat diet for 8 weeks. In vitro *E. faecium* R0026 showed an ability to lower the low-concentration of cholesterol by 46%, and the ability to lower the highconcentration of cholesterol by 58%. LCBE significantly reduced the body weight gain, Lee index, brown fat index and body mass index of mice on a high-fat diet. Moreover, LCBE markedly improved serum lipids (including serum triglyceride, total cholesterol, low-density lipoprotein and highdensity lipoprotein) while also significantly reducing liver total cholesterol. Serum lipopolysaccharide and total bile acid in L and H groups decreased significantly compared with M group. PCR-DGGE analysis showed that the composition of gut microbiota in the treatment groups was improved. *Akkermansia muciniphila* was found in H group. The PCA result indicated a similar gut microbiota structure between LCBE treatment groups and normal group while the number of bands and Shannon diversity index increased significantly in the LCBE treatment groups. Finally, qPCR showed *Bifidobacterium* spp. increased significantly in H group compared with M group, LCBE alleviated liver steatosis and improved brown adipose tissue index.

## Introduction

Obesity has become a major health problem [1]. It is closely associated with the development of metabolic syndrome (MetS), including hyperlipidemia, type 2 diabetes, insulin resistance and non-alcoholic fatty liver, which are all important risk factors that lead to cardiovascular disease [2]. Obese individuals have greater susceptibility to elevated total cholesterol (TC) with some differences between men and women [3]. Obesity is closely related to the occurrence of hyperlipidemia and the typical dyslipidemia seen in obesity is usually manifested by an increase in triglyceride (TG) concentration. A decrease in high-density lipoprotein cholesterol (HDL-C) concentration is accompanied by HDL dysfunction and low-density lipoprotein cholesterol (LDL-C) concentration is normal or slightly increased [4]. Brown adipose tissue (BAT) helps with weight control and has anti-obesity effects [5].

Recent studies have pointed to a relationship between gut microbiota and body weight. The gut microbiota in humans plays a role in weight gain [6], and shows differences in overweight individuals [7], as some of the bacteria are involved in microbial homeostasis and weight loss [6].

A great deal of evidence has revealed that probiotics work to inhibit intestinal infections and maintain gut microbiota balance [8] while also exerting anti-obesity activities. Diet-induced obese mice showed a reduction in body weight gain after supplementation with *Lactobacillus rhamnosus* PL60 [9]. Kang *et al*. found that *Lactobacillus gasseri* BNR17 significantly lowered body weight and fat pad mass in diet-induced overweight rats [10].

*Enterococcus faecium* (*E. faecium*) is one of the normal bacteria in the human gut [11]. Soy yoghurt supplemented with *E. faecium* was found to have a lipid-lowering effect on rats with hypercholesterolemia [12]. Isolation of *E. faecium* from rhizospheric soils can remove cholesterol in vitro [13]. *Enterococcus faecalis* FK-23 (FK-23) exhibited an anti-obesity effect in HFD-fed mice [14].

*Bacillus subtilis* (*B. subtilis*) administration restored a healthy balance of beneficial and harmful bacteria in the gut [15]. *B. subtilis* ATCC 6633 affected the composition or function of the commensal, bacterial and host epithelia. Furthermore, it also restricts bacterial and lipopolysaccharide (LPS) translocation [16]. *B. subtilis* R0179 survives passage through the human GI tract and is well tolerated by healthy adults [17]. B. subtillis KC-3 fermented soymilk (F-KC) decreased lipid content in 3T3-L1 adipocytes by inhibiting lipogenesis and showed anti-obesity activities [18].

The safety evaluation of live combined *B. subtilis* R0179 and *E. faecium* R0026 (LCBE) used in Asia has found that LCBE has little risk for consumers [19]. The mixed culture of *E. faecium* and *B. subtilis* significantly increases the number of viable bacteria more so than using one strain alone (Patent No. CN104371958 A). LCBE orally or alone (1.0×10^8^ CFU/kg/day) for 3 days was able to normalize the Treg/CD4+ ratio in the blood and mesenteric lymph nodes in a female Wistar rat model. LCBE also normalized the smooth muscle contractility in post-inflammation rats [20]. Moreover, LCBE adhered to human intestinal (HT-29) cells in culture and exerts a bacteriostatic effect on a number of enteric pathogens including *E. coli*, *Shigella flexneri*, *Salmonella enterica* serotype Typhi, *Staphylococcus aureus*, and *Candida albicans* [21, 22].

This study aimed to evaluate the influence of LCBE on obesity-associated hyperlipidaemia and gut microbiota in C57BL/6 mice.

## Materials and Methods

### Preparation of LCBE

*B. subtilis* R0179 and *E. faecium* R0026 were from probiotic product Medilac-S (Hanmei Pharmaceuticals, China), which was composed of 4.4 × 10^9^ colony-forming units (CFU) of *B. subtilis* R0179 and 6.0 × 10^10^ CFU of *E. faecium* R0026.

### Bacterial Strains and Cultivation

*E. faecium* R0026 was activated three times by aseptic Mann Rogosa Sharpe (MRS) medium (Beijing Aoboxing Bio-tech Co., Ltd.), and the facultative anaerobes were incubated at 37°C for 24 h.

### Analysis of Cholesterol-Lowering Ability of *E. faecium* R0026 In Vitro

Cholesterol alcohol solution with a mass concentration of 15 mg/ml was added to the culture medium. A cholesterol-medium without uninoculated bacterial was used as a control sample. *E. faecium* R0026 was inserted at a 10% inoculation volume. Samples were taken at 12, 15, 18, and 21 h and centrifuged at 1,000 g/min for 10 min. Then, the supernatant was collected to determine the concentration of cholesterol (TC Detection Kit, Nanjing Jiancheng Bioengineering Institute, China) [23].

### Animals and Diets

**Fig. 1:**
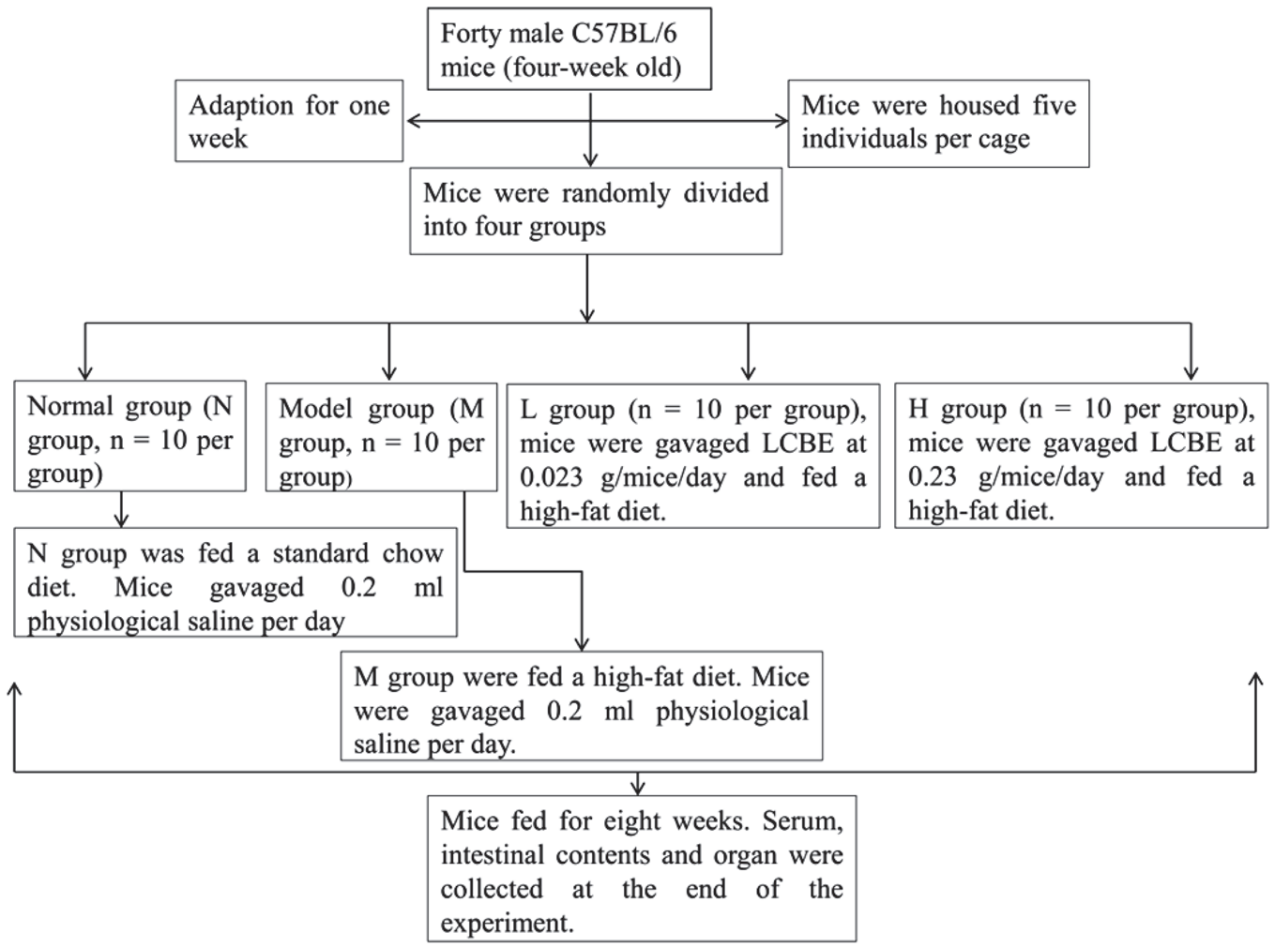
The animal experimental protocol.

Forty male C57BL/6 mice (four-week-old) were bought from Dalian Medical University. Mice were housed five individuals per cage and bred in pairs in an SPF laboratory animal house (Model Animal Research Centre, Dalian Medical University, China). Mice were given free access to food and water. After adaptation for one week, mice were randomly divided into four groups (*n* = 10 per group), normal group (N), model group (M) and two dosage groups (L group and H group). The L group was gavaged LCBE at 0.023 g/mice/day and H group was gavaged LCBE at 0.23 g/mice/day. LCBE was dissolved in physiological saline. The N group was fed a standard chow diet (D12450, Research Diets Inc., USA), the treatment groups and M group received a high-fat diet (Beijing HuaFuKang Bioscience, China). N and M groups were gavaged 0.2 ml physiological saline per day. Mice were gavaged with LCBE and fed with high-fat diet for 8 weeks, until the average body weight of the model group was 20% of the normal group, reaching the obesity standard. The animal experimental protocol was shown in Fig. 1. The research was approved by the Committee for Animal Ethics of Dalian Medical University (AEE18037).

### Analysis of Body Weight Gain, BMI and Organ Index

The weight of mice was measured once a week. Body mass index (BMI) was measured by [body weight (g)/length^2^ (cm^2^)]; Lee’s index was measured by body length (anal–nasal length)/body weight, multiplied by 100%; liver index was measured by wet weight of liver/body weight, multiplied by 100%; epididymal fat index was measured by wet weight of the epididymal fat/body weight, multiplied by 100%; brown fat index was measured by wet weight of interscapular BAT/body weight, multiplied by 100% [24]. An Accu-Chek Performa (ROCHE, Germany) blood glucose meter was used to monitor blood glucose levels by measuring tail veins in mice.

### Analyses of Serum and Liver Biochemical

The serum was placed on ice and melted naturally. Serum was mixed in a vortex shaker for 30 sec. Serum TC and liver TC were measured via the glycerol cholesterol oxidase-peroxidase. Total cholesterol concentration = (sample OD - Blank OD)/(calibrator OD- Blank OD) × calibrator concentration [25]. Serum TG and liver TG were measured via the glycerol phosphate oxidase-peroxidase using the same method as with TC and test kit from Nanjing Jiancheng Bioengineering Institute. Next, a visible light homogeneous phase removal method based on the selective action of mixed surfactants and a chemiluminescence homogeneous phase removal method were used to determine serum HDL and LDL [25]. Serum total bile acid (TBA) was measured by the enzymatic colorimetric method. (TBA concentration = ΔA determination/ΔA standard × standard concentration (μmol/l))[26]. The serum LPS was measured by enzyme-linked immunosorbent assay using the relevant ELISA kits (Shanghai Langdun Biological Technology Co., Ltd., China). The above index was tested by microplate reader (Thermo, USA).

### Pathologic Evaluation

The liver and BATs were fixed in 10% formaldehyde. The tissues were then embedded in paraffin, sectioned into 5 μm thick slices and stained by hematoxylin-eosin (HE). Stained sections were photographed at 100× and 10× magnifications by applying a light microscope (Nikon Instruments CO., Ltd., Japan) [27].

### DNA Extraction from the Intestinal Contents

Gut microbiota genomic DNA was extracted from 0.1 g frozen cecal contents per sample by the Stool DNA Isolation Kit (Foregene, China). DNA was eluted with 100 μl of Elution Buffer provided in the kit. Microbial DNA concentration and purity were assessed by spectrophotometry. DNA was stored at −80°C.

### Analysis of Gut Microbiota

Bacterial 16S rRNA genes were amplified using universal primers, the forward 338f GC (5'-CGC CCG CGC GCG GGC GGG GCG GGG GCA CGG GGG GAC TCC TAC GGG AGG CAG CAG-3') and the reverse 518r (5'-ATT ACC GCG GCT GG-3') targeting the V3 region. PCRs were conducted in a total volume of 50 μl systems, 25 μl of Master Mix, 1 μM 338f and 518r primers, and 20 μl of RNase-free water. Three microliters of DNA was used as a template. PCR conditions: 94°C for 5 min, then 94°C for 30 sec, annealing at 54.5°C for 30 sec and extension at 72°C for 1 min by 30 cycles, followed by a final extension step at 72°C for 7 min. PCR products were visualized on a 2% agarose gel followed by denaturing gradient gel electrophoresis (DGGE) analysis. PCR products were analyzed by DGGE for microbiota and separated by DCode System according to the instructions of the manufacturer [28].

Next, the amplicons were applied to 8% (w/v) polyacrylamide and 1× TAE (40 mM Tris-acetate, 20 mM sodium acetate, 1 mM EDTA) with a denaturant gradient of 30-60%. Gels were run at 60°C for 6 h at a constant voltage of 83V in 1x TAE buffer. The nucleic acids in the gels were then stained in 1× TAE buffer containing EB nucleic acid stain (Solarbio Science & Technology Co., Ltd., China) for 30 min, and then the gel was imaged with a ChemiDocTM XRS system (Bio-Rad, USA) and analyzed using Bio-Rad Quantity One Software [29].

### Excision and Sequencing Analysis of Bands

Some special individual bands were cut from the gel and placed in a centrifuge tube containing 20 μl of distilled water and incubated at 4°C overnight. The cut bands were amplified by 16S primers without GC clips, and the amplified products were examined by agarose gel electrophoresis. Then, these PCR amplicons were sent to General Biological Systems Co., Ltd. (Anhui, China) for purification and sequencing. Finally, the sequences were compared with those deposited with GenBank at the National Center for Biotechnology Information using the basic BLAST search tools (https://blast.ncbi.nlm.nih.gov/Blast.cgi?PAGE_TYPE=BlastSearch&BLAST_SPEC=MicrobialGenomes ).

### Quantification of Dominant Gut Microbiota by Real-Time PCR

Quantitative real-time PCR was performed with the 3005p instrument (Agilent, USA) and SYBR Green chemistry (Trans Gen Biotech, China). The copy numbers of *Bifidobacterium* spp., *Escherichia coli* subgroup, *Lactobacillus* spp. were analyzed and the primers of real-time PCR are shown in Table 1 [30-32]. PCR reaction includes a total of 10 μl, 5 μl of SYBR Master Mix, 0.2 μl of each primer, 0.2 μl of passive Reference Dye (50×), 1 μl of DNA as template, and 3.4 μl of water). PCR conditions are as follows: 30 sec at 94°C followed by 30 cycles at 94°C for 5 sec, 60°C for 30 sec at the appropriate annealing temperature. The specific 16S rRNA primers amplifying genes melting curve analysis of products was used to confirm that fluorescence signal originated from the specific amplification product. The relative genes expression of gut microbiota normalized to 16S rRNA expression and internal reference in fecal samples using the comparative CT (threshold cycle) method [33]. All results were normalized and calculated by using the 2^−ΔΔCT^ method.

### Statistical Analysis

Values were presented as mean and standard deviation and all statistical analyses were performed by using SPSS version 20 (IBM, USA). Graph Pad Prism Version 5 (Graph Pad Software Inc, USA) was used to analyze and process data. The DGGE bands analysis used Quantity One software (Bio-Rad). The real-time PCR data were analyzed by MxPro-Mx3005P System and software (Agilent, USA). The unpaired Student’s t-test was used to compare two groups and one-way analysis of variance was used to compare the varieties between all groups. A statistically significant probability value was *p* < 0.05.

## Results

### Effect of *E. faecium* R0026 on High Cholesterol

*E. faecium* R0026 has a cholesterol-lowering effect in cholesterol medium. The cholesterol-lowering ability was between 46%-58% (Supplementary Material 1).

### LCBE Reduced Body Weight Gain and Body Mass Index and Improved Organ Index in Obesity Mice

**Fig. 2:**
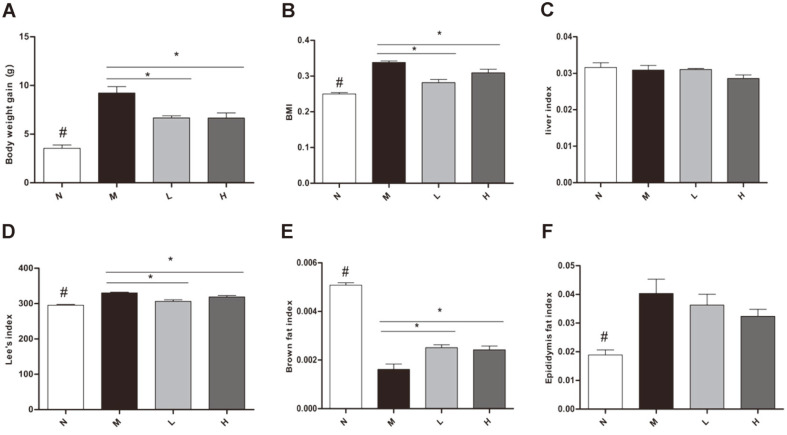
Effect of LCBE in body weight gain, BMI, Lee’s index, liver index, epididymal fat index and brown fat index. (**A**) Body weight gain. (**B**) Body mass index (BMI). (**C**) Liver index. (**D**) Lee’s index. (**E**) Brown fat index. (**F**) Epididymal fat index. N, normal group; M, model group; L, low-dose group; H, high-dose group. #*p* < 0.05 the N vs. the M group, *p < 0.05 vs. the M group. #*p* < 0.05 the N vs. the M group, *p < 0.05 vs. the M group.

Body weight gain in L and H groups reduced significantly compared with M group (Fig. 2A). BMI in L and H groups reduced significantly compared with M group (Fig. 2B). The Lee’s index (Fig. 2D) and brown fat index (Fig. 2E) in L and H groups reduced significantly compared with M group (Fig. 2D). The liver index was not altered between groups (Fig. 2C). Comparing with N group, epididymal fat index in M group increased significantly, but there were no differences in treatment groups compared with M group (Fig. 2F). Blood glucose level in N group reduced significantly, but there were no differences in treatment groups compared with M group (Supplementary Material 2).

### Effect of LCBE on Obesity-Induced Hyperlipidemia

**Fig. 3:**
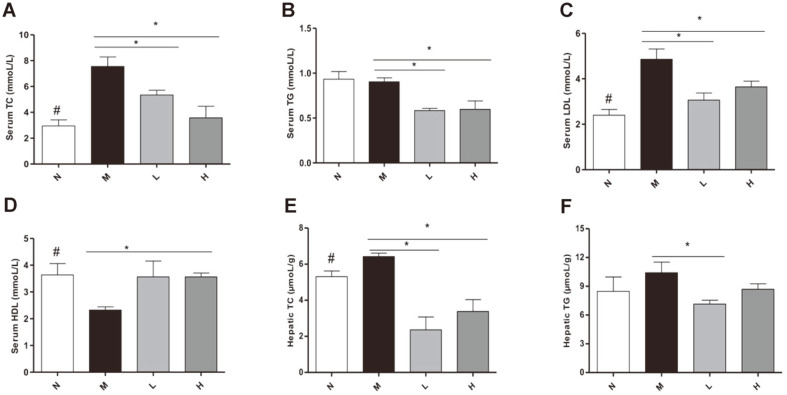
Effect of LCBE in lipid levels. (**A**) Serum TC. (**B**) Serum TG. (**C**) Serum LDL. (**D**) Serum HDL. (**E**) TC in liver. (**F**) TG in liver. N, normal group; M, model group; L, low-dose group; H, high-dose group. #*p* < 0.05 the N vs. the M group, *p < 0.05 vs. the M group. #*p* < 0.05 the N vs. the M group, *p < 0.05 vs. the M group.

LCBE reduced serum TC, TG, and LDL levels in L and H groups compared with M group (Figs. 3A-C). The levels of HDL in serum increased in the H group compared with M group (Fig. 3D). Liver TC reduced significantly in L and H groups compared with M group (Fig. 3E) and liver TG in N group showed no significant difference compared with M group (Fig. 3F).

### Effect of LCBE on Obesity-Associated Inflammation

Analysis revealed that serum LPS and total TBA in M group increased significantly compared with N group, while in L and H groups the same levels were reduced significantly compared with M group (Supplementary Material 3).

### Effects of LCBE on Gut Microbiota by PCR-DGGE and Real-Time PCR

**Fig. 4:**
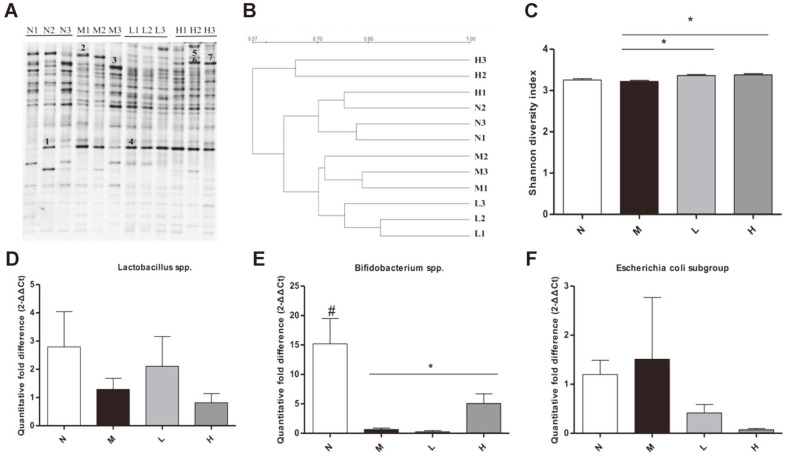
Effect of LCBE in gut microbiota. DGGE gel profile constructed by primer of 16S rRNA gene (**A**) bacterial population. (**B**) A cluster analysis (UPGMA) of the DGGE profiles. (**C**) The bacterial diversity analysis by Shannon diversity index. (**D**) The copy number of *Lactobacillus* spp. species. (**E**) The copy number of the *Bifidobacterium* spp. species. (**F**) The copy number of *Escherichia coli* subgroup species. N, normal group; M, model group; L, low-dose group; H, high-dose group. #*p* < 0.05 the N vs. the M group, *p < 0.05 vs. the M group.

PCR-DGGE results showed differences in cecum microbiota between normal, model and treatment groups. DGGE gel profiles showed highly different distribution of gut microbiota in different groups (Fig. 4A). A cluster analysis (UPGMA) of the DGGE profiles revealed results in two separated groups. M group samples clustered together as a one separated group, N group and treatment group’s samples clustered together as another separated group. Moreover, H group was clustered more closely to N group, H group gut microbiota showed a great similarity with N group (Fig. 4B). PCA data showed treatment groups demonstrate a good separation of samples. L and H groups’ samples were more similar to N groups’ samples (Supplementary Material 4). The band number (Supplementary Material 4) and Shannon diversity index in L and H groups increased significantly compared with M group (Fig. 4C). Evenness was not altered among the four groups (Supplementary Material 5).

From real-time PCR, M group showed a significant decrease in *Bifidobacterium* spp. compared with N group, and it was significantly higher in H group than in M group (Fig. 4E). *Lactobacillus* spp. (Fig. 4D) and *Escherichia coli* *subgroup* (Fig. 4F) were not altered among the four groups.

### Sequence Analysis of Cut Dominant DGGE Bands

The most representative 8 bands were excised from gel and subjected to 16s rRNA sequencing. Two band sequences were identified at species level using a 97% similarity threshold. The band 2 in M group was identified as *Alistipes shahii* with 99.21%. Band 6 was identified as *Akkermansia muciniphila* (*A. muciniphila*) with 98.01% in H group.

### Histological Effects of LCBE on Liver and Brown Adipose Tissue

**Fig. 5:**
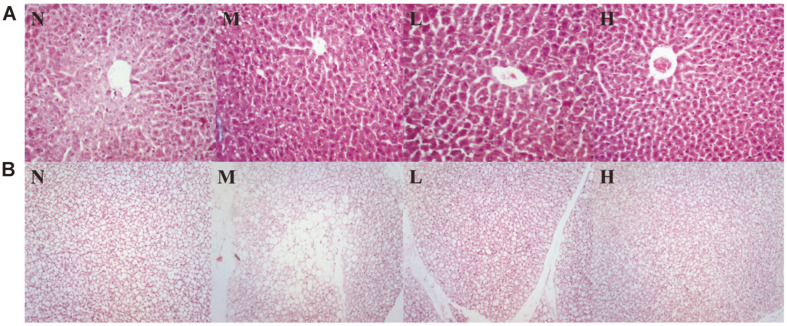
Effects of LCBE on liver and brown adipose tissue assessed by H&E staining. Liver tissue representative images are at 100× magnification and brown adipose tissue representative images are at 10×. N, normal group; M, model group; L, low-dose group; H, high-dose group.

From HE staining, liver histology showed more steatosis in M group compared with N group, while hepatic steatosis in liver was significantly improved after LCBE treatment in L and H groups. In brown adipose tissue, M group showed fat accumulation was accompanied by a significant increase in vacuole formation compared with N group. L and H groups significantly improved compared with M group (Fig. 5).

## Discussion

Obesity is a common metabolic disorder that is closely related to lipid metabolism disorders and systemic inflammation [34]. The cholesterol assimilation ability is of particular interest with probiotics. Probiotics improve obesity and its metabolic syndrome. High-fat diet-induced obese mice and rats fed with *Lactobacillus* and *Bifidobacterium* decreased body weight, and decreased fat tissue weight in the abdominal area. Insulin resistance, metabolic syndrome, inflammation or liver steatosis were improved [35-37]. Bagci *et al*. found that *E. faecium* isolated from human milk or colostrum can absorb cholesterol to varying degrees. Its cholesterol lowering ability is between 25.2%-64.1% [38]. In this study, *E. faecium* R0026 absorbed cholesterol to varying degrees. The cholesterol-lowering ability was between 46%-58%.

LCBE administration suppressed body weight gain, BMI and ectopic lipid accumulation in liver on the obese mice. *E. faecalis* can produce myristoleic acid (which an unsaturated SCFA). *E. faecalis* and its metabolites can activate BAT and reduce obesity [39]. In our study BAT index in Land H groups increased significantly compared with M group. Fermented soymilk with *B. subtilis* KC-3 led to a decrease in levels of leptin secretion and an increase in levels of glycerol secretion in the 3T3-L1 adipocytes. In addition, mRNA expression levels of both SREBP-1c (sterol regulatory element-binding protein 1-c) and PPAR-gamma (peroxisome proliferator-activated receptor-gamma) in cells treated with F-KC markedly downregulated [18]. Another study showed LCBE treatment reduced alcohol-induced hepatic histopathological injury in alcoholic liver disease (ALD), while also reducing serum TG, aspartate aminotransferase (AST), endotoxin, D lactic acid levels and serum inflammatory factor levels in rats. LCBE modulated gut microbiota in rats with ALD [40]. In this study, parameters related to dyslipidaemia (TC, TG, LDL) were reduced by LCBE. High-dose LCBE significantly improved the HDL. *E. faecalis* FK-23 upregulated the expression levels of genes involved in fatty acid oxidation in the liver tissue to attenuate hepatic steatosis in the liver of HFD-fed mice [14]. Liver TC and hepatic steatosis in LCBE treatment groups reduced significantly compared with M group in our research.

LCBE and glutamine ameliorated ALD by suppressing inflammation and regulating the gut microbiota [40]. *B. subtilis* species secrete a cyclic peptide with antibacterial potential and thus may downregulate expression of pro-inﬂammatory cytokines to exert their anti-inﬂammatory effects [41]. In our study, LCBE treatment decreased the level of LPS and total TBA in serum, indicating the metabolic endotoxemia decreased in obese mice. Furthermore, LCBE attenuated inflammation-mediated liver damage.

In this research, differences in cecum microbiota among the normal, model and treatment groups were observed. A cluster analysis (UPGMA) of the DGGE profiles revealed that LCBE improved gut microbiota composition in obese mice induced by high-fat diets. PCA results showed significant differences in the natural composition and structure of the gut microbiota between M group and treatment groups. The Shannon diversity index in L and H groups increased significantly compared with M group. L and H groups significantly increased the number of bands compared with M group. The result showed that LCBE could modulate the gut microbiota composition to reverse metabolic disorders in C57BL/6 obese mice. Recently, several reports pointed to the interaction between BAT and gut microbiota [39]. Our result showed that L and H groups significantly improved fat accumulation in BAT compared with M group by HE staining.

The numbers of *Bifidobacterium* spp. in H group were significantly higher than in M group. Similar results were reported in resveratrol treatment increasing the growth of *Lactobacillus* and *Bifidobacterium* in obesity mice [42]. *A. muciniphila* was identified from H group bands. According to a previous study, *A. muciniphila* was a potential new probiotic against obesity. *A. muciniphila* produced SCFAs and had a negative correlation with total body weight gain [43]. We found *Alistipes shahii* in M group. *Alistipes shahii* produced bacterial sulfurlipids (SLs) in the cecum of mice. SLs were significantly increased in mice that were fed high-fat diets [44].

## Conclusions

Our results demonstrated that live *Bacillus subtilis* R0179 and *Enterococcus faecalis* R0026 reversed obesity in mice. There is a great interest for identifying novel probiotics with anti-obesity function. Next, we will use the fecal microbiota transplantation (FMT) method to verify whether these two probiotics achieve anti-obesity effects due to the improvement of the gut microbiota. In summary, our findings provide a basis for probiotics in the treatment of obesity-related diseases.

## Supplemental Materials



Supplementary data for this paper are available on-line only at http://jmb.or.kr.

## Figures and Tables

**Table 1:** Primers of quantitative real-time PCR.

qPCR specificity	Primer	Sequence (5’-3’)	Annealing temperature
*Escherichia coli* subgroup	ECO-F	GTTAATACCTTTGCTCATTGA	61°C
	ECO-R	ACCAGGGTATCTAATCCTGTT	
*Bifidobacterium* spp.	Bifid-F	CTCCTGGAAACGGGTGG	55°C
	Bifid-R	GGTGTTCTTCCCGATATCTACA	
*Lactobacillus* spp.	Lac-F	AGCAGTAGGGAATCTTCCA	58°C
	Lac-R	CACCGCTACACATGGAG	
