# Supplementary material for: Enterococcus faecium R0026 Combined with Bacillus subtilis R0179 Prevent Obesity-Associated Hyperlipidemia and Modulate Gut Microbiota in C57BL/6 Mice
Source: J Microbiol Biotechnol. 2020 Oct 20;31(2):181–8. doi: 10.4014/jmb.2009.09005 (PMC9706029; doi:10.4014/jmb.2009.09005)
Supplement: Supplementary file 1 [file jmb-31-2-181-supple.pdf]

J. Microbiol. Biotechnol.
https://doi.org/10.4014/jmb.2009.09005

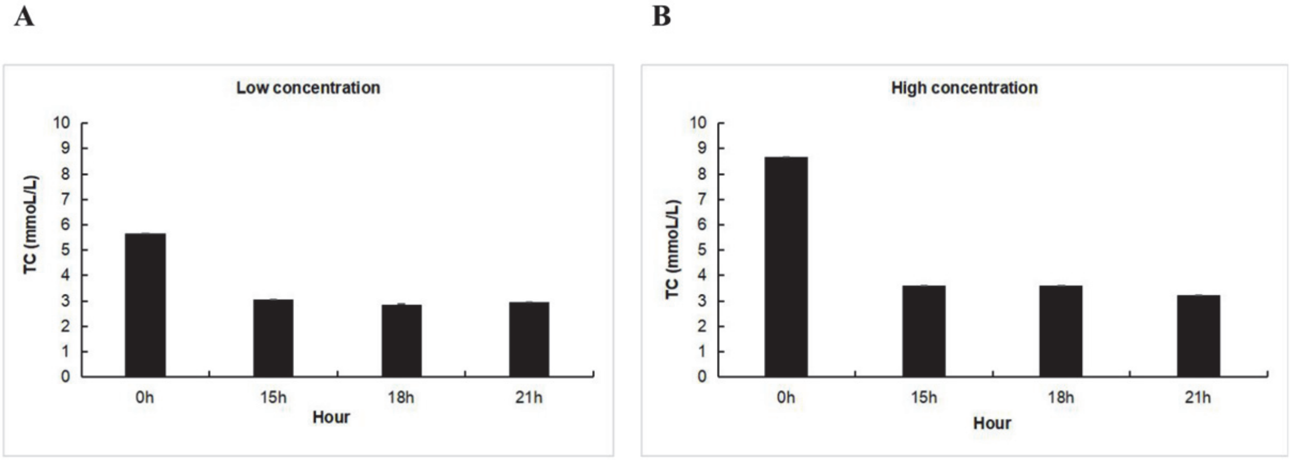

Supplementary material. 1. Effect of *Enterococcus faecium* R0026 on high cholesterol in vitro. (A) Low concentration. (B) High concentration.

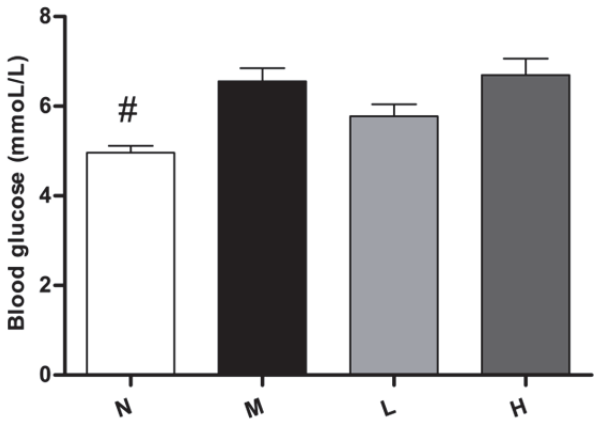

Supplementary material. 2. Effect of LCBE in blood glucose. N, normal group; M, model group; L, low dose group; H, high dose group. $^{\#}P < 0.05$ the N vs. the M group, $^{*}P < 0.05$ vs. the M group.

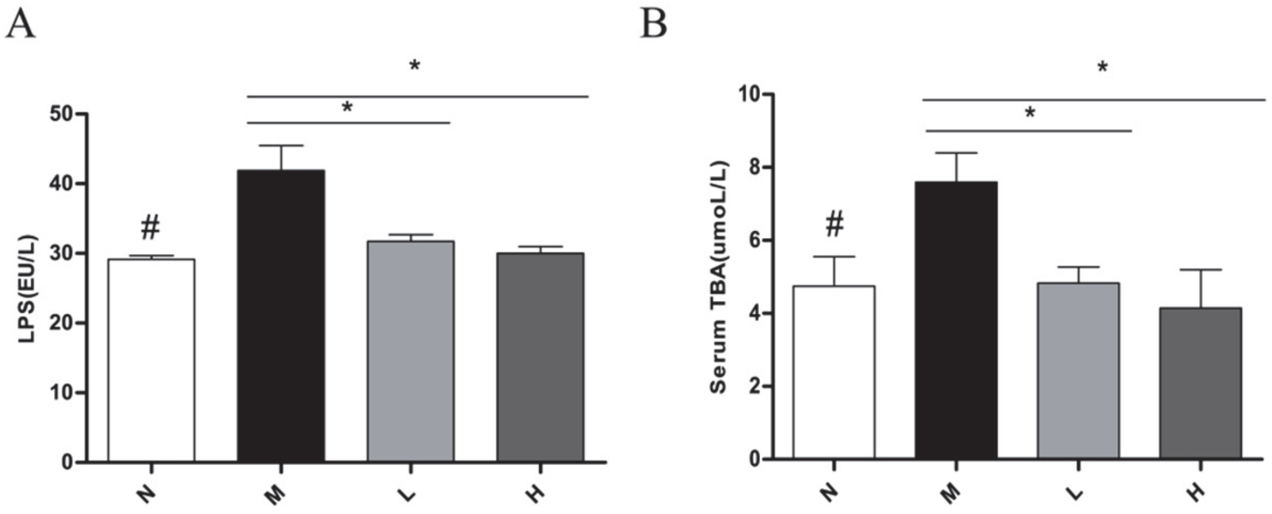

Supplementary material. 3. Effect of LCBE in serum inflammatory markers. (A) Serum lipopolysaccharide (LPS). (B) Serum total bile acid (TBA). N, normal group; M, model group; L, low dose group; H, high dose group. #*P* < 0.05 the N vs. the M group, **P* < 0.05 vs. the M group.

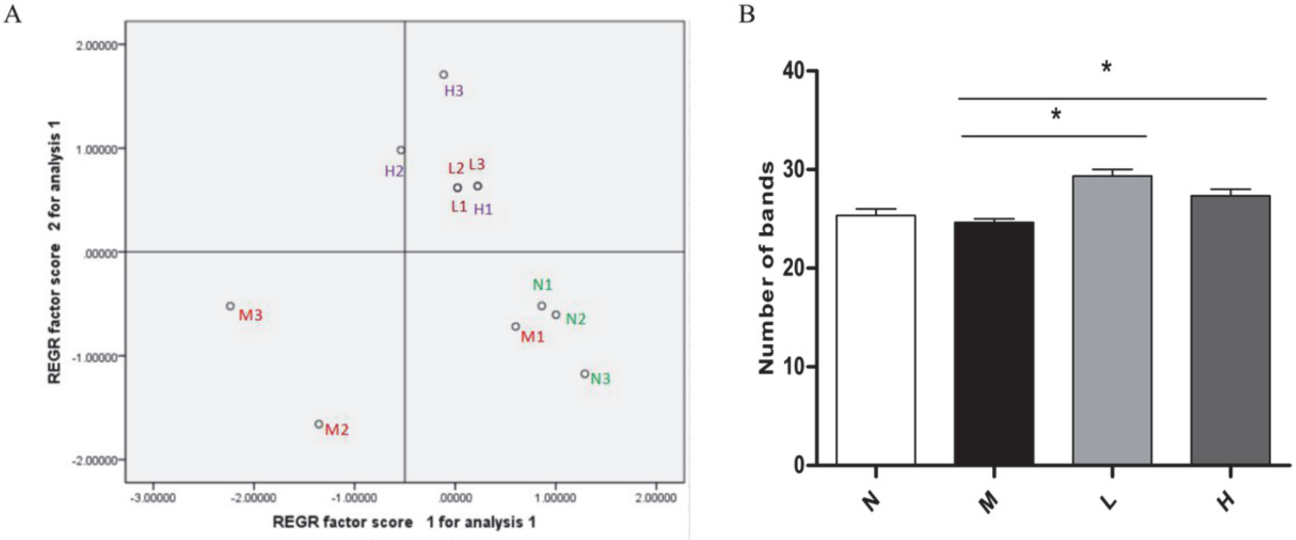

Supplementary material. 4. Effect of LCBE in gut microbiota. (A) PCoA of 16S rRNA profiles. (B) Gut microbiota richness measures, number of DGGE bands. N, normal group; M, model group; L, low dose group; H, high dose group. #*P*< 0.05 the

N vs. the M group, $^{*}P < 0.05$ vs. the M group.

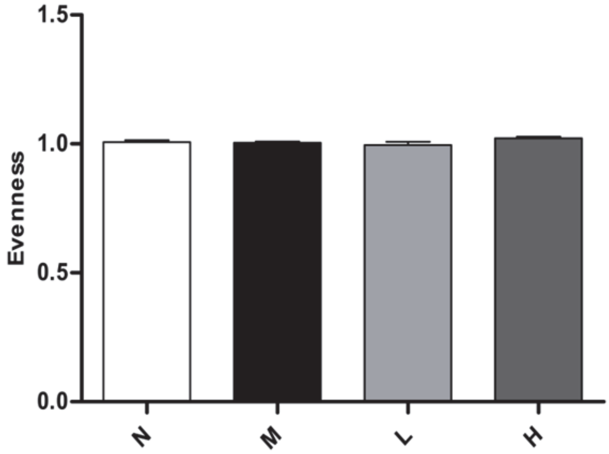

Supplementary material. 5. Effect of LCBE in gut microbiota. PCR-DGGE analysis the evevness of the gut microbiota. N, normal group; M, model group; L, low dose group; H, high dose group. $^{\#}P< 0.05$ the N vs. the M group, $^{*}P < 0.05$ vs. the M group.
